# Minimally invasive gas embolization using acoustic droplet vaporization in a rodent model of hepatocellular carcinoma

**DOI:** 10.1038/s41598-019-47309-y

**Published:** 2019-07-30

**Authors:** Jennifer N. Harmon, Foad Kabinejadian, Robinson Seda, Mario L. Fabiilli, Sibu Kuruvilla, Cathleen C. Kuo, Joan M. Greve, J. Brian Fowlkes, Joseph L. Bull

**Affiliations:** 1 Department of Biomedical Engineering, Tulane University, New Orleans, Louisiana USA; 2 Data Office for Clinical and Translational Research, University of Michigan, Ann Arbor, Michigan USA; 3 Department of Radiology, University of Michigan, Ann Arbor, Michigan USA; 4 Department of Oncology, Stanford University, Stanford, California USA; 5 Department of Neuroscience, Tulane University, New Orleans, Louisiana USA; 6 Department of Biomedical Engineering, University of Michigan, Ann Arbor, Michigan USA

**Keywords:** Targeted therapies, Biomedical engineering

## Abstract

Hepatocellular carcinoma is the third leading cause of cancer-related deaths worldwide. Many patients are not eligible for curative therapies, such as surgical resection of the tumor or a liver transplant. Transarterial embolization is one therapy clinically used in these cases; however, this requires a long procedure and careful placement of an intraarterial catheter. Gas embolization has been proposed as a fast, easily administered, more spatially selective, and less invasive alternative. Here, we demonstrate the feasibility and efficacy of using acoustic droplet vaporization to noninvasively generate gas emboli within vasculature. Intravital microscopy experiments were performed using the rat cremaster muscle to visually observe the formation of occlusions. Large gas emboli were produced within the vasculature in the rat cremaster, effectively occluding blood flow. Following these experiments, the therapeutic efficacy of gas embolization was investigated in an ectopic xenograft model of hepatocellular carcinoma in mice. The treatment group exhibited a significantly lower final tumor volume (ANOVA, p = 0.008) and growth rate than control groups – tumor growth was completely halted. Additionally, treated tumors exhibited significant necrosis as determined by histological analysis. To our knowledge, this study is the first to demonstrate the therapeutic efficacy of gas embolotherapy in a tumor model.

## Introduction

Liver cancer is the third most common cause of cancer-related death globally; in addition to the high mortality rate, incidence rates are continuing to rise^1,2^. Patients with hepatocellular carcinoma (HCC), the most common type of liver cancer, are faced with a poor prognosis. The majority of patients are unable to receive curative therapies such as a liver transplant or surgical resection of the tumor, and HCC exhibits minimal response to systemic chemotherapy^3,4^. Instead, many patients receive non-curative therapies to extend their life or downstage the disease^5–7^. Transarterial chemoembolization (TACE), one such non-curative therapy, is the gold standard for patients with intermediate stage HCC^1,8^. TACE is the combination of a technique known as bland transarterial embolization (TAE), which utilizes an intraarterial catheter to deposit an embolizing agent directly into the vasculature of a tumor, with local delivery of a chemotherapeutic agent. The intent of these therapies is to induce targeted ischemia and subsequent tumor necrosis due to lack of blood flow. Both TAE and TACE increase survival rate and stall tumor progression^6,9^. TAE and TACE leave much room for improvement, however. They require arterial access and have some restrictive guidelines for patient qualification, including relatively healthy overall hepatic function and a lack of portal venous thrombosis. These are necessary due to potential off-target effects produced by the induction of large scale arterial ischemia^5,7^. Gas embolotherapy (GE) has been proposed as a less invasive, highly spatially selective embolization method, with the intention of circumventing the issues presented by TAE and TACE^10,11^. GE utilizes focused ultrasound (FUS) and a phenomenon known as acoustic droplet vaporization (ADV) to produce gas emboli directly within a tumor’s vasculature.

ADV involves the conversion of a perfluorocarbon droplet into a microbubble under exposure to FUS. A number of diagnostic and therapeutic applications have been proposed. The bubbles generated by ADV are highly echogenic and can be used as imaging contrast agents. Molecular imaging applications, which utilize the inclusion of a targeting ligand in the droplet and subsequently the bubble’s stabilizing shell, have also been investigated^12,13^. For drug delivery or gene therapy applications, the droplet can be loaded with a drug or genetic material which can be released in a target area (e.g., a tumor) upon vaporization^14–19^. GE, another proposed therapeutic application, involves the accumulation of the microbubbles generated by ADV within the vasculature of a tumor. Droplets can be produced with a mean diameter of 1–3 μm, which enables them to flow freely through capillaries; upon vaporization, the droplets undergo a 125-fold volumetric expansion, rendering them large enough to embolize small vessels^20^. They can also be targeted to specific cell receptors (e.g., α_v_β_3_) to increase their local concentration, thereby increasing the likelihood of forming a large embolus during insonation, and to increase the stability of a developed occlusion by increasing its resistance to dislodging. In addition to targeting the droplets themselves using a receptor-ligand interaction, FUS can be targeted non-invasively with high spatiotemporal resolution, allowing for the localized induction of an embolus without requiring that the embolizing particles be deposited directly into the tumor vasculature. The droplets can be administered through a simple intravenous injection or infusion, thereby eliminating the need for arterial access.

We have previously performed extensive modeling of GE, primarily with regards to bubble expansion and transport in the vasculature, to elucidate the physical underpinnings of the treatment *in silico* and in benchtop experiments^21–29^. Additionally, two previous studies have been performed to better understand how GE may perform *in vivo*, and one recent study utilized an *ex vivo* model. The first used GE to reduce renal perfusion in canines via occlusion of the renal artery^30^. The second was performed to visually identify the configuration of lodged bubbles in the rat cremaster muscle in order to determine if the *in vivo* results were consistent with experiments previously performed in polydimethylsiloxane (PDMS) models; the *ex vivo* study also identified the configuration of lodged bubbles in addition to documenting some bioeffects^31–33^. The first portion of the study currently being reported utilizes the rat cremaster muscle as an environment for real-time optical imaging of ADV *in vivo*, with the primary objective of inducing and visually observing substantial vaporization and occlusion in individual vessels. The lengths and diameters of the resulting occlusions are measured. The study confirms the feasibility of producing large, stable gas emboli and of fully occluding microvessels in the rat cremaster. ADV-induced occlusion in a murine model of HCC is then examined. A complete cessation of tumor growth was observed, thereby rendering this the first study, to our knowledge, to demonstrate the therapeutic efficacy of GE in a tumor model.

## Results

### Rat cremaster embolization and bioeffects

**Figure 1:**
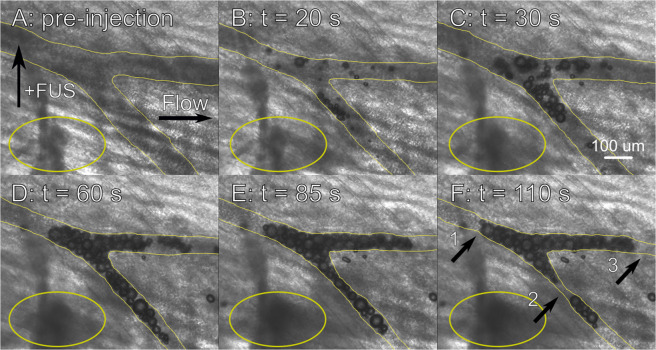
Vaporization and occlusion in an auxiliary artery in the rat cremaster. (**A**) Before injection of droplet solution, under exposure to ultrasound (7.5 MHz, 2.95 MPa, 8 voltage cycles, 100 Hz PRF). Ultrasound alone produced no discernable effect. The focal spot of the ultrasound (+FUS) and the flow direction are denoted with arrows. (**B–F**) 20, 30, 60, 85, and 110 seconds after the injection of droplet solution, respectively. Substantial vaporization occurred, leading to a complete cessation of flow in the upper branch and substantial flow reduction in the lower branch of the vessel. The observed cessation of blood flow occurred between 30 (**C**) and 60 (**D**) seconds. The vessel is traced in yellow; vasoconstriction occurs as the embolus develops. The sections of the vessel most affected by vasoconstriction upstream of the bifurcation, in the lower branch of the vessel, and in the upper branch of the vessel are marked by arrows 1, 2, and 3 respectively. This specific vessel exhibited a −68.14% change in vessel diameter in the most affected section of the lower branch (arrow 2). Additionally, vaporization-induced hemorrhage is highlighted by the yellow ellipses; it appears as a dark, expanding cloud. Scale bar = 100 µm.

Intravital microscopy experiments were performed using the rat cremaster to assess the therapeutic feasibility of GE by documenting substantial ADV-induced vascular occlusion and any associated bioeffects. These experiments expanded on the work reported in Samuel *et al*.^31^, which primarily served to provide the first optical evidence of ADV-induced occlusion *in vivo* and to determine if the configuration of lodged bubbles *in vivo* were consistent with experiments previously performed in PDMS models^32^. In each animal (N = 5), substantial vaporization and occlusion were observed in either the feeder vessel or auxiliary vessels in the cremaster muscle. Figure 1 shows an example of vaporization and occlusion in an auxiliary artery in the cremaster over the course of 110 seconds. The vessel is outlined in yellow. Ultrasound was initiated prior to injecting droplet solution as a negative control (Fig. 1A). A bolus injection of droplet solution was administered shortly before the image in Fig. 1B; the gas embolus can be seen developing in both branches of the artery in Fig. 1C–F. Complete cessation of blood flow, as determined by the lack of movement of red blood cells, was achieved in the upper branch of the artery between 30–60 seconds after the injection of droplet solution and maintained until at least 110 seconds (Fig. 1C,D,F). Flow was reduced, but not entirely blocked in the lower branch; a portion of the embolus had begun to separate from the primary embolus after approximately 1 minute (Fig. 1D–F). The initial vessel diameters in this example were 91 μm upstream of the bifurcation, 87 μm in the lower branch of the bifurcation, and 66 μm in the upper branch. The diameters were 56 μm (Fig. 1F, arrow 1), 28 μm (Fig. 1F, arrow 2), and 32 μm (Fig. 1F, arrow 3) following embolization, respectively.

One notable bioeffect that was observed during ADV was vasoconstriction; this is most noticeable in the lower branch of the vessel in Fig. 1F (arrow 2). On average, vessels were 136 ± 7 μm in diameter prior to embolization and exhibited a 53.7 ± 2.71% reduction in diameter after the occlusion developed (N = 21). The average length of the emboli was 274 ± 39 μm. In addition to vasoconstriction, hemorrhage was observed in the tissue surrounding the embolized vessels. The yellow oval in Fig. 1 highlights a region of visible hemorrhage, which appears as a darker gray cloud-like region that expands over the course of the ultrasound exposure. Hemorrhage was consistently observed in the vicinity of the vessels in which vaporization and embolization occurred, while vasoconstriction was noted primarily in larger vessels (76–195 μm initial diameter). Notably, in each observed case of vessel occlusion, a number of bubbles accumulated to form a composite embolus rather than coalescing into a single, large bubble. This differed from results previously observed in benchtop experiments^32^ and the previous cremaster study conducted by Samuel *et al*.^31^, which reported long, sausage-like occlusions comprised of single bubbles. These results served as confirmation of the feasibility of highly-localized, ADV-induced embolization and the ability to deliver droplets through an intravenous injection.

### Treatment efficacy and bioeffects in the tumor model

**Figure 2:**
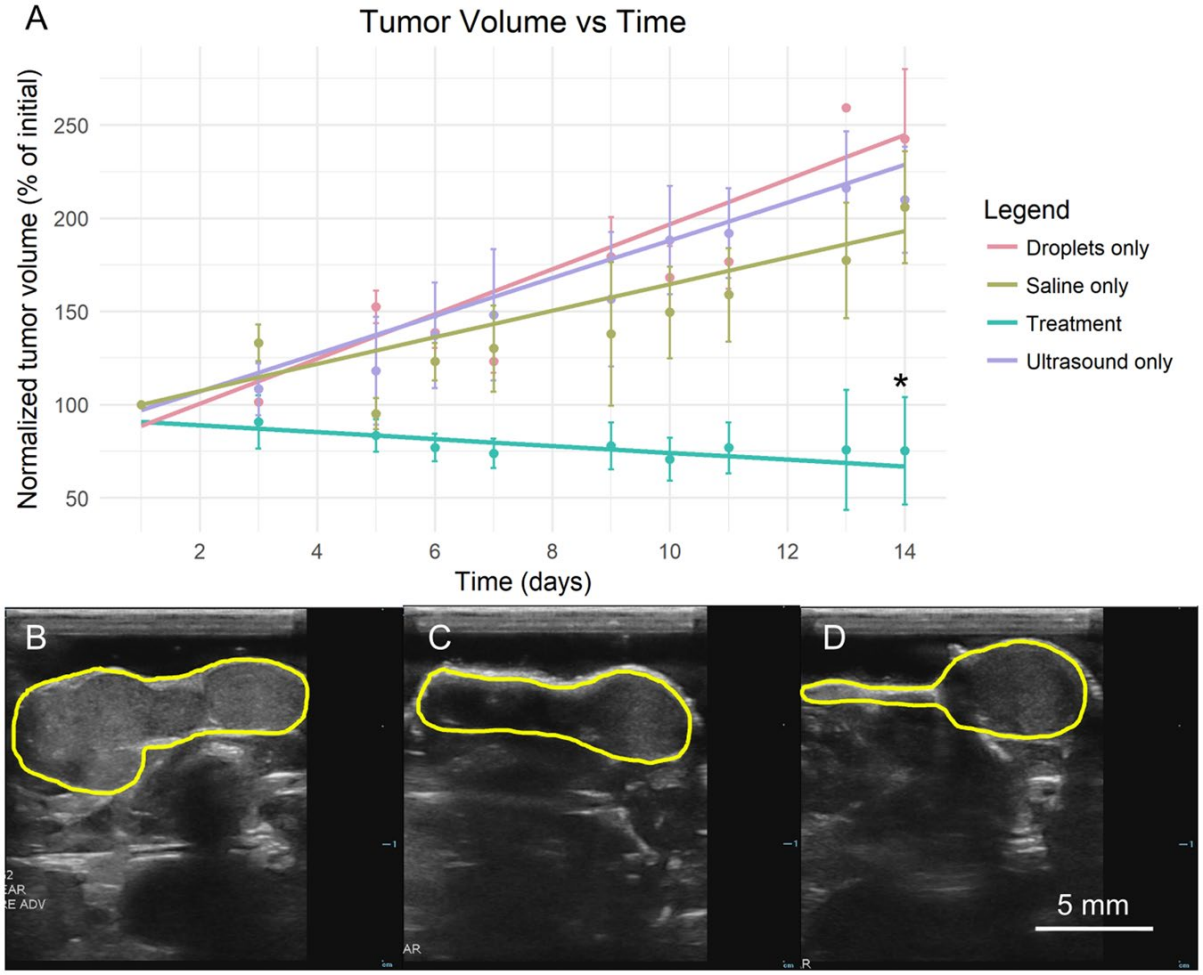
Tumor volume over the two-week treatment course. (**A**) Normalized tumor volume is plotted with respect to time. Error bars representing one standard error and the linear regression lines used for the growth rate comparisons are shown on the plot. The treatment group had a significantly lower final volume than controls (*p = 0.008, ANOVA, N = 5 per group). The treatment group also exhibited a complete cessation of growth; the slope of the linear regression line was not significantly different from zero (p = 0.069). The growth rate in the treatment group was significantly lower than in the droplets only (p < 0.001), saline only (p = 0.016), and FUS only (p = 0.005) control groups as determined by linear regression. (**B–D**) B-mode images of a representative treated tumor are shown at Days 1, 6, and 14, respectively. This particular tumor exhibited a reduction in tumor volume, with the final volume being 37.6% relative to the initial volume. Scale bar = 5 mm.

Following the confirmation of feasibility in the cremaster experiments, the treatment was tested in a murine model of HCC. Tumor volume was measured as the primary indicator of efficacy over the two-week treatment course. Figure 2A illustrates the changes in tumor volume over time for each group. The final tumor volume in the treatment group (75.34 ± 28.8% of the initial volume) was significantly lower than the droplets only (242.5 ± 37.6%), saline only (206.1 ± 30.0%), and FUS only (210.0 ± 28.4%) control groups (ANOVA, p = 0.008, N = 5 for each group). There were no significant differences in the final tumor volume between the control groups (ANOVA, p = 0.688). To examine the effect of the treatment on the tumor growth rate, linear regression models were generated for each group using the tumor volume data. These are plotted in Fig. 2A. The estimates of the slopes and the corresponding 95% confidence intervals given by the models were used to compare the tumor growth rate between groups. The tumor growth rate in the treatment group (−1.85 ± 2.00% of the initial tumor volume per day) was significantly lower than the droplets only (12.0 ± 2.98% per day, p < 0.001), saline only (7.13 ± 3.66% per day, p = 0.016), and FUS only controls (10.1 ± 4.17% per day, p = 0.005) and was not significantly different from zero (p = 0.069). This indicated that tumor growth was completely halted. The tumor growth rate was not significantly different between control groups, indicating that neither droplet injections nor FUS exposure alone affected the tumor growth rate as compared to a saline-only control (p = 0.850 and 0.294, respectively). Figure 2B–D provides B-mode ultrasound images of a tumor in the treatment group at Days 1, 6, and 14. This particular mouse exhibited tumor regression, presenting with a final tumor volume of 37.6% relative to the initial volume.

**Figure 3:**
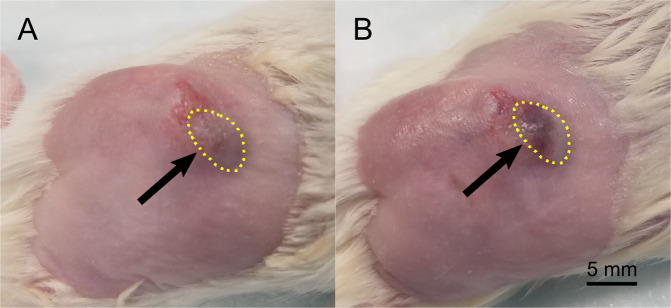
Hemorrhage on and distal to the surface of the skin in a treated tumor. Images are shown of the tumor before (**A**) and after (**B**) treatment on Day 5. Hemorrhage is observed following treatment; this is visible as a dark purple region on the surface of the skin in (**B**). The tumor is outlined with a dotted yellow line. The hemorrhagic region is highlighted with an arrow. Scale bar = 5 mm.

Bioeffects were observed during the treatment. Varying degrees of apparent hemorrhage occurred in each animal of only the treatment group, visually observed on and distal to the surface of the skin. This can be seen in Fig. 3. In all cases this progressed to a scab by the end of the treatment course.

### Histological analysis

**Figure 4:**
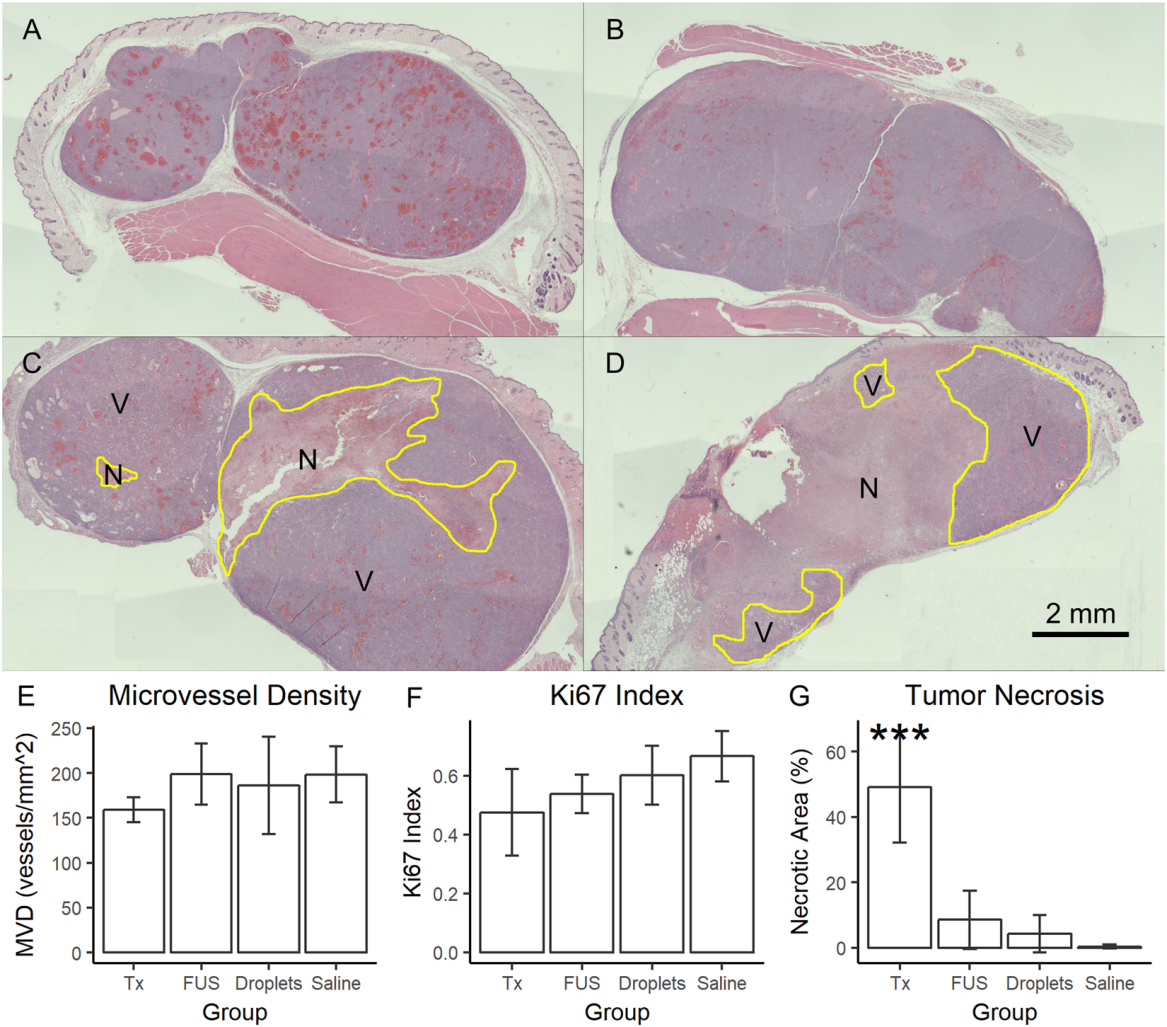
Histological analysis of treated and control tumors. (**A–D**) Stitched images of H&E stained saline only (**A**), droplets only (**B**), ultrasound only (**C**), and treated (**D**) tumors taken at 2× magnification. Necrosis appears as a lighter pink, whereas the viable tumor is a darker purple. Necrotic and viable tumor sections are separated by a yellow line; an N denotes necrotic tissue, whereas a V denotes viable tissue. The ultrasound only tumor in this image is 18.24% necrotic while the treated tumor shown is 66.02% necrotic; each represents the highest necrotic percentage from their respective groups. The treated tumor pictured here is the remaining right half of the tumor pictured in D. (**E–G**) Quantitative results are plotted for each group for MVD, Ki67 index, and necrosis percentage. No significant differences were observed for MVD or Ki67 index, but the treated tumors had a significantly higher necrosis percentage. ***p < 0.001, ANOVA. Scale bar = 2 mm.

At the end of the two weeks of treatment, the mice were euthanized and the tumors were harvested for histological analysis. H&E staining provided general morphological information about the tumors following treatment. Representative images of H&E stained tumors, as well as the quantitative results from the analysis of stained tissue sections, can be seen in Fig. 4. Most notably, treated tumors exhibited widespread necrosis; this was quantified as a percent of the tissue section that was comprised of necrotic tissue as compared to the overall area of tumor tissue in the section. The percent of the tumor that was necrotic was significantly higher in the treated tumors (49.2 ± 7.63%) as compared to ultrasound only (8.53 ± 4.02%), droplets only (4.30 ± 3.29%), and saline only (0.37 ± 0.37%) controls (ANOVA, p < 0.001). While it appears there is a slight trend, with FUS alone potentially inducing a small amount of necrosis, there were no significant differences between the control groups (ANOVA, p = 0.200).

Immunohistochemical procedures were used to evaluate the MVD and proliferative potential of the tumors following treatment. Analysis was performed on non-necrotic areas of the treated tumors to determine if the remaining, non-necrotic tumor maintained its capacity for growth and survival, or if the treatment had a moderate therapeutic benefit that was sub-threshold for the induction of necrosis. MVD was quantified following CD31 staining while proliferative potential was quantified using the Ki67 index. It was determined that neither the MVD nor the Ki67 index were significantly different between treatment and control groups (ANOVA, p = 0.368 and 0.139, respectively), suggesting that the remaining tumor is viable on Day 14.

## Discussion

The average occluded vessel diameter in the cremaster experiments was significantly greater than in the previous cremaster-based GE study (53 ± 11 μm vs. 36 ± 1 μm, t-test, p = 0.04)^31^. The maximum occluded vessel diameter (241 μm) was also substantially higher than the maximum reported by Samuel *et al*.^31^ (<65 μm). Additionally, the current study visually confirmed the ability to form occlusions by vaporizing intravenously administered droplets, whereas the previous study required arterial delivery of the droplets through a catheter placed in the carotid artery of the rats. These results are promising, indicating that GE may be able to induce large scale occlusion within a tumor in a less invasive manner than both the previous study and TAE, the current clinical method of embolic delivery. While these results were observed in some animals following the initial bolus injection of droplet solution, others required multiple injections. It is reasonable to infer that the animals that received multiple injections had carryover droplets that remained in the bloodstream, given that subsequent injections were administered within 5–10 minutes of the initial bolus, thereby increasing the effective droplet concentration within the target vasculature with each injection. This suggests that the ability to consistently embolize large vessels may be partially dependent on the concentration of droplets. This may serve as one explanation for the increase in the scale of embolization observed in the current study as opposed to Samuel *et al*.^31^. Although a similar concentration of droplets was used in the previous work (10^8^ droplets/mL, 1 mL per animal vs. 10^8^–10^9^ droplets/mL, 0.2–1 mL per animal in the current study), the droplet shell was comprised of bovine serum albumin. The droplets used in the current study were produced with a PEGylated lipid shell; PEGylation is widely used to reduce the clearance of liposomes by the mononuclear phagocyte system^34^. We posit that this effect increased the effective droplet concentration in the target vasculature by reducing droplet clearance, thereby leading to more vaporization and larger occlusions. Future studies may seek to elucidate the pharmacokinetic behavior of the droplets used in the current study and to optimize the administered concentration of droplet solution.

Both vasoconstriction and hemorrhage were observed either in the vessel being occluded or in surrounding capillaries. Hemorrhage is likely caused by droplets vaporizing within smaller capillaries; droplets on the order of 1–2 µm in diameter will expand to bubbles of 5–10 µm upon vaporization, as compared to a capillary diameter of 4–7 µm in the rat cremaster^35^. Given the relatively rapid and violent nature of the phase transition, as evidenced by previous studies aimed at elucidating the underlying mechanism of ADV and the subsequent bubble expansion, and the large size of the droplets and bubbles with respect to the capillary diameter, the vessel walls will be subjected to large shear and intramural stresses^28,36–40^. This is consistent with predictions made in our previous computational and theoretical studies, which predicted that ADV would produce large wall stresses, potentially inducing endothelial damage^25,28^. Capillaries, comprised of a single layer of endothelial cells, will be prone to rupture, leading to the extravasation of red blood cells as seen in Fig. 1. Vasoconstriction occurred in larger, multi-layered vessels, with a smooth muscle layer supporting the endothelial cells. While these vessels do not experience the same severity of ADV-induced shear and intramural stress due to their larger size compared to the droplets and bubbles, it is likely that some smaller-scale vaporization-induced endothelial damage was occurring. While high-intensity ultrasound alone has previously been shown to induce vasoconstriction or degeneration of the tunica media^41,42^, bioeffects were not observed until after the injection of droplet solution. Additionally, other studies have been published that observed endothelial cell damage in response to ultrasonically-driven microbubble contrast agents^43–45^, and we have previously experimentally observed ADV-induced endothelial damage^46^. Given that endothelial cell damage has been shown to lead to vasoconstriction^47^, it is likely that ADV-induced damage of the vessel wall is responsible for the observed bioeffects. Despite the potential issues that may be associated with hemorrhage and vasoconstriction, both of these bioeffects are likely beneficial in the context of ischemia induction when induced in a highly spatially targeted manner. Hemorrhage reduces the number of viable, blood-carrying vessels within a given area while vasoconstriction during ADV facilitates the formation of emboli.

Following the visual confirmation of the feasibility of occluding large vessels using intravenously administered droplets in the cremaster experiments, the treatment was tested in a murine model of HCC. Tumor growth was completely halted and a large portion of the remaining tumor was necrotic. Given that partial necrosis has been correlated with a positive tumor response in patients with HCC^48^, this is a promising result. A cessation of disease progression and the associated extension of patient life may be crucial for patients on the waitlist for a liver transplant, a potentially curative therapy for non- or intrahepatic metastatic HCC. Additionally, pre-transplant TAE has been associated with a reduced recurrence rate of HCC following transplant; it is reasonable to infer that GE would produce similar effects^6,49,50^.

The RGD targeting ligand used in the current study was included to maximize the therapeutic potential of GE by increasing the local concentration of droplets within the tumor, thereby increasing the probability of forming large emboli. Ectopic xenograft tumors comprised of HepG2 cells have previously been shown to overexpress the integrin α_v_β_3_; targeting approaches to this receptor have shown some promise for both diagnostic and therapeutic applications with microbubbles^51,52^. The utility of the targeting ligand is not directly demonstrated in the current study, however. The potential therapeutic benefit conferred by the inclusion of RGD on the droplet shell as compared to untargeted droplets should be investigated in future studies.

Though the current treatment method shows promise in terms of its efficacy, it also induced off-target effects, including the hemorrhage observed in the skin superficial to the tumor. This was likely primarily due to the superficial nature of the subcutaneous tumor model; this led to ADV within the skin itself given that the target was relatively small. The hemorrhage and skin damage generated by this off-target ADV were likely exacerbated over the treatment course by the occasional administration of a depilatory cream. Future plans include the use of nude mice to circumvent the necessity of hair removal and controlled placement of the ultrasound focus to avoid further superficial ADV. It is important to note, however, that the induction of hemorrhage at the ultrasound focus is not inherently an issue – if confined to the tumor, it could assist in starving the tumor of its blood supply. Anti-vascular therapy has previously proven effective in a murine tumor model^53^, and may enhance the effects of GE. In addition, previous work in the literature has indicated that neither the induction of intratumoral hemorrhage nor the exposure of a tumor to the relatively low amplitude FUS utilized in this study increases the probability of tumor metastasis^53,54^, thereby indicating that the added therapeutic benefit of the bioeffects discussed here will likely outweigh any potential negative effects. This should be investigated explicitly in future studies, however, by monitoring for metastasis during treatment.

Future studies will focus on maximizing the efficacy of the therapy. One area for improvement is the method of insonation; if the entire tumor is uniformly insonated, it may be possible to achieve tumor regression. The current study utilized a manually-guided single element ultrasound transducer equipped with a coupling cone for treatments. While this provided rapid and facile treatment administration, it also introduced a number of uncontrollable variables in terms of the distribution of the delivered ultrasound. The H&E stained sections that demonstrated a non-contiguous boundary between necrotic and viable tissue are indicative of inconsistent vaporization and embolization throughout the tumors. The CD31 and Ki67 staining results served as further confirmation that the entire tumor needs to be treated, given that the remaining viable tumor in the treated mice displayed no reduction in MVD or proliferative potential, although the CD31 results may be influenced by the time point at which the animals were sacrificed (Day 14) given that there will be some lag time between vessel occlusion and the vessel pruning and regression process^55^. We are currently prototyping an ultrasound-guided vaporization method to increase our control over the placement of the ultrasound focus while maintaining the ease and speed of treatment administration. In addition to utilizing a uniform insonation method, the current treatment may be combined with targeted chemotherapy delivery using drug-loaded droplets, moving the treatment from a TAE analog to a TACE analog, allowing us to match the current clinical gold standard^1,8^. These improvements may produce a better tumor response and allow for less frequent treatments, a crucial aspect for the clinical translatability of this method.

In conclusion, GE is a feasible method of targeted vessel occlusion, as demonstrated in the rat cremaster. Although the proposed therapy induces bioeffects, such as vasoconstriction and hemorrhage, these will likely prove beneficial in the context of ischemia induction in a tumor. GE halted growth and induced necrosis in treated tumors. Future improvements may include the use of an ultrasound-guided method of insonating the tumor and concurrent targeted drug delivery to reduce the amount of viable tumor remaining after treatment. GE shows promise as a minimally-invasive, easily and quickly administered treatment for HCC, with the potential to become an ultrasound-guided, ultrasound-induced, and ultrasound-monitored therapy.

## Methods

### Droplet preparation

A lipid film was prepared by combining the following lipids dissolved in chloroform: 1,2-distearoyl-sn-glycero-3-phosphocholine (DSPC, 80 mol%, Avanti Polar Lipids, Alabaster, AL, USA), 1,2-distearoyl-sn-glycero-3-phosphoethanolamine-N-[methoxy(polyethylene glycol)-2000] (ammonium salt) (DSPE-mPEG2000, 10 mol%, Avanti Polar Lipids), and 1,2-distearoyl-sn-glycero-3-phosphoethanolamine-N-[maleimide(polyethylene glycol)-2000] (ammonium salt) (DSPE-mPEG2000-Mal, 10 mol%, Avanti Polar Lipids). The lipids were dried under vacuum to remove the chloroform. The resulting lipid film was hydrated using a diluent consisting of phosphate buffered saline (80% v/v, Life Technologies, Grand Island, NY, USA), propylene glycol (10% v/v, Sigma-Aldrich, St. Louis, MO, USA), and glycerol (10% v/v, Sigma-Aldrich) and heated to 70 °C. The final concentrations of DSPC, DSPE-mPEG2000, and DSPE-mPEG2000-Mal in the lipid blend were 2, 0.95, and 0.95 mg/mL, respectively. The lipid blend was combined, in a 3:1 volumetric ratio, with perfluoropentane (PFP, Strem Chemicals, Newburyport, MA, USA). The phases were emulsified on ice using a sonicator operated at 36 µm amplitude for 15 seconds (Q55, 20 kHz, 55 W, QSonica, Newton, CT, USA). The resulting emulsion was washed, via repeated centrifugation, to remove unincorporated lipids, and characterized with a Coulter counter (Multisizer 4, Beckman Coulter, Brea, CA, USA). A representative sample of droplets was measured at 1.22 ± 0.66 µm diameter. An RGD peptide (c(RGDfC), Peptides International, Louisville, KY, USA) was coupled to the droplets via a maleimide linkage to the DSPE-mPEG2000-Mal to target the integrin α_v_β_3_, a receptor known to be overexpressed in HCC models similar to the one used in the current study^52^.

### Rat preparation

The method of rat preparation was adapted from Samuel *et al*.^31^. All animal procedures were conducted with the approval of the Institutional Animal Care and Use Committee (IACUC) and in accordance with animal use guidelines specified by the Unit for Laboratory Animal Medicine at the University of Michigan. A detailed explanation of the procedure is provided in the Supplementary Methods. In brief, the cremaster muscle of male Sprague-Dawley rats (Charles River Laboratories, Wilmington, MA) was exposed using blunt dissection and separated from the testicle following catheterization of the tail vein. The animal was placed into a custom acrylic tank containing an optical window over which the cremaster muscle was splayed. The cremaster was stretched radially and held in place by sutures wrapped around pins placed around the optical window. The tank was then filled with degassed normal saline and placed on an inverted microscope.

### Optics and acoustics, cremaster

**Figure 5:**
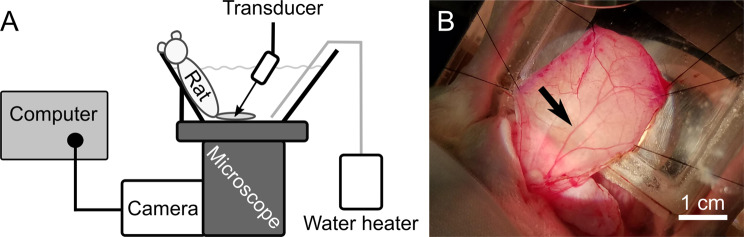
FUS and imaging setup for the cremaster experiments. Following the surgical exposure of the cremaster muscle, the rat was placed into a custom-made acrylic tank. The cremaster muscle was splayed over an optical window at the bottom of the tank before placing the tank onto an inverted microscope to allow for real-time optical imaging within the cremaster. (**A**) A side view is shown of the rat in the tank on the inverted microscope with its cremaster muscle stretched over the optical window. The transducer was positioned such that the full cremaster thickness was placed within the full width at half maximum of the transducer focus in the axial direction. The transducer was laterally positioned to place the focus in the center of the viewing frame recorded by the camera, such that images and video could be captured at the vaporization site. The microscope stage was used to move the animal to focus on different sections of the cremaster. Warmed saline was added for acoustic coupling and the maintenance of osmotic balance in the cremaster; the temperature was maintained at 37 °C by a therapeutic heat pump (water heater). The camera used to record videos and capture images, as well as the computer used to interface with the camera, are shown in the left portion of the figure. (**B**) A top-down photographic image of the cremaster before insonation is shown. The cremaster is stretched over the optical window and held in place by sutures. The feeder vessel of the cremaster muscle is marked with a black arrow. Scale bar = 1 cm.

A truncated schematic side view of the FUS and imaging setup for the cremaster experiments is shown in Fig. 5. A waveform generator (33120 A, Agilent, Santa Clara, CA, USA) created a square wave, which gated a sinusoidal wave generated by a second function generator (3314 A, Hewlett-Packard, Palo Alto, CA, USA). The output from the function generator was amplified by a gated amplifier (60 dB, GA-2500, Ritec, Warwick, RI, USA) before being output to the transducer. The voltage waveforms sent to the transducer were monitored using an oscilloscope (Wavesurfer 44MXs-A, Teledyne LeCroy, Chestnut Ridge, NY, USA). The pulse repetition frequency (PRF) of the ultrasound was controlled using a 20 MHz sweep function generator (BK Precision 4040 A, BK Precision Corporation, Yorba Linda, CA, USA).

A single-element 7.5 MHz transducer with a diameter of 1.9 cm, a focal length of 3.81 cm and a lateral full width half maximum of approximately 420 μm (A321S, Olympus Panametrics-NDT, Waltham, MA) was used. The electronic setup applied 8 cycle voltage pulses, generating 2.95 MPa peak rarefactional pressure as measured in the free field using a fiber optic probe hydrophone^56^. The peak negative pressure was selected such that substantial ADV and the formation of gas occlusions were observed during the experiments without visually observing subsequent destruction of the bubbles comprising the emboli *in situ*. The PRF was varied between 100–1000 Hz. A Point Grey camera (Flea 3, FLIR Integrated Imaging Solutions, Vancouver, Canada) was used for image and video capture in conjunction with Point Grey FlyCap2 image capture software (2.7.3.0, FLIR Integrated Imaging Systems, Vancouver, Canada).

### Rat experimental design

Animal preparation was performed as described above. The ultrasound focus was positioned to the center of the viewing frame of the camera. The acoustic parameters are described in the previous section. Ultrasound exposure was initiated prior to the injection of droplet solution. An initial 0.2 mL bolus of droplet solution (10^8^–10^9^ droplets/mL) was delivered intravenously; video capture was initiated shortly before injection and continued until vaporization or embolization occurred. Following this initial injection, 0.1 mL injections were delivered every 5–10 minutes to attempt to induce more vaporization in the feeder vessel or in auxiliary vessels in the cremaster, with a maximum volume of 1 mL of droplet solution being administered during an experiment. After each ultrasound exposure, the tissue was scanned and pictures of any occluded vessels near vaporization sites were captured. The maximum duration of the experiment was 4 hours, including the time taken to prepare the animals. At the end of the experiment, each animal was humanely euthanized according to IACUC guidelines.

### Tumor inoculation in mice

HepG2, an HCC cell line, was obtained from the American Type Culture Collection (ATCC, Manassas, VA, USA). Cells were cultured in 500 mL MEM supplemented with 10% FBS, 1% antimycotic antibiotic solution, 1% minimum non-essential amino acids, 1% sodium pyruvate, and 1 mL gentamicin; all media and additives were from Gibco Laboratories (Gaithersburg, MD, USA). Cells were cultured at 37 °C in air supplemented with 5% CO_2_.

All animal procedures were conducted with the approval and guidance of the IACUC at Tulane University. NOD SCID Gamma mice (NSG, 6–8 weeks old, Jackson Laboratory, Bar Harbor, ME, USA) were maintained in accordance with Department of Comparative Medicine guidelines at Tulane University. A subcutaneous injection of 5 million HepG2 cells in 150 µL of a 1:1 mixture of Matrigel (Matrigel^TM^ Matrix, Corning Inc., Corning, NY, USA) to media was administered on the flank of the mouse. Tumor size was monitored using calipers; tumor volume was calculated using the formula V = ½ (length)(width)^2 ^^57^. Tumor progression was monitored for 6–8 weeks before assigning a mouse to one of the following groups: (i) droplet solution injection and ultrasound exposure (treatment), (ii) droplet solution injection only, (iii) ultrasound exposure only, or (iv) saline injection only (N = 5 per group). The average initial tumor volume was 238.4 ± 7.0 mm^3^; the initial tumor volume was not significantly different between groups (Kruskal-Wallis, p = 0.912).

### Treatment and imaging

The tumors were insonated using a 2.5 MHz, single element transducer (H-108, Sonic Concepts, Bothell, WA, USA). Prior to reaching the transducer, the amplifier output was passed through an impedance matching network (3^rd^ Harmonic Impedance Matching Network, Sonic Concepts, Bothell, WA, USA). The remainder of the electronic setup is identical to that used in the cremaster experiments. The ultrasound was operated at 2.5 MHz using 13 cycle pulses (5.391 µs pulse width) which generated a 5.34 MPa peak negative pressure, and a PRF of 100 Hz. The ultrasound system and parameters were modified from those in the cremaster experiments to increase the likelihood of inducing substantial vaporization in the thicker tumor, where tissue-induced attenuation of the ultrasonic signal would play a greater role. The peak negative pressure, pulse width, and PRF used for treatments in the current study were substantially lower than acoustic parameters used in previously reported ADV-assisted HIFU experiments, such that substantial tissue heating and thereby thermal ablation would not occur during treatment^58^. A coupling cone was attached to the transducer, filled with degassed water, and covered with Tegaderm (3M, Maplewood, MN, USA), an acoustically-transparent membrane.

Droplet solution was administered via a tail vein injection prior to anesthetizing the animals (125 µL, 3.5 × 10^6^ droplets/µL). The mice were induced at 2–3% isoflurane in oxygen gas (1 L/min) and maintained between 1–1.5% isoflurane in oxygen during the experiment. Artificial tears were placed on the eyes to prevent desiccation. Mice were kept on a heated pad (Gaymar T/Pump and T/Pad, Stryker, Kalamazoo, MI, USA) during the experiments to maintain their core body temperature. Ultrasound images of the tumor were taken prior to and following FUS exposure. A Zonare ultrasound system (ZS3, Mindray Medical International Limited, Shenzhen, China) with a linear array transducer operated at 25 MHz (L25–8 linear array, Mindray Medical International Limited) was used for the ultrasound images. The tumor area was insonated for 2 minutes with the equipment and parameters described above within 10 minutes of the injection of droplet solution. The treatment duration was selected based on the results from the cremaster experiments, in which fully-developed occlusions were visually observed following 30–60 seconds of insonation (Fig. 1C,D). The insonation time was increased to two minutes to allow for treatment of the tumor, a larger target than vessels in the cremaster muscle. During the insonation, the transducer was held by an operator and manually scanned over the surface of the tumor to attempt to treat the entire lesion. The total duration of the procedure was less than 30 minutes per mouse. Mice were treated in cycles of three consecutive days followed by one day off for two weeks. This resulted in a total of 11 treatment days and 3 rest days. The animals were sacrificed on Day 14. Three control groups were tested as well. Phosphate buffered saline was substituted for the droplet solution in the ultrasound only and saline only groups and the ultrasound exposures were not performed on the droplets only and saline only groups. Following the treatment course, animals were humanely euthanized according to appropriate IACUC guidelines.

### Histology

Following euthanasia, tumors were harvested for histological analysis. A detailed explanation of the procedures is provided in the Supplementary Methods. In brief, 5 µm thick sections of formalin-fixed, paraffin embedded tumor tissue underwent either hematoxylin and eosin (H&E) staining or immunohistochemical procedures to detect CD31 and Ki67 using polyclonal rabbit anti-mouse primary antibodies (ab28364 and ab15580 respectively, Abcam, Cambridge, United Kingdom) and an Abcam EXPOSE Rabbit specific HRP/DAB detection IHC kit (ab80437). CD31 and Ki67 staining were performed to quantify microvessel density (MVD) and proliferative potential (Ki67 index) respectively. Sections were imaged using a color Point Grey camera (Blackfly S, FLIR Integrated Imaging Solutions, Vancouver, Canada) on an inverted microscope (Eclipse Ti2, Nikon, Tokyo, Japan) at 40× magnification.

Analysis of the slides was done in a blinded manner. H&E stained slides primarily revealed the presence of necrotic tissue within treated tumors; this was quantified using ImageJ (Rasband, W.S., ImageJ, U. S. National Institutes of Health, Bethesda, Maryland, USA). A freehand ROI was drawn over the necrotic region of the tumor and the pixel area was compared to that of an ROI drawn over the entire tumor section, thereby giving a percent of the tumor that was necrotic. For both cell proliferation and MVD, a “hot spot” counting method was used, in which image regions containing the highest staining density were selected for analysis^59,60^. For the treated tumors, hot spots were selected only within the remaining viable tumor, excluding the necrotic region. Five images were selected from each tissue section. The Ki67 index was defined as the percentage of nuclei that stained positive for Ki67. The MVD was defined as the number of microvessels, identified by positive CD31 staining, per unit area.

### Statistical analysis

All descriptive statistics are presented as mean ± standard error except for the representative droplet sizes given above, which are mean ± standard deviation. R was used for all statistical analysis^61^. A one-way ANOVA with a Tukey post-hoc test was used for comparing the final tumor volume between the treatment and control groups. For comparing tumor volumes between treatment and control groups over a time course, a linear regression model was generated. The 95% confidence interval of the slope of each regression line was calculated; Equation 4 from Paternoster *et al*.^62^ was used to calculate a p-value for the comparisons between treatment and control groups. A Kruskal-Wallis test was performed to confirm that initial tumor volumes did not significantly vary between groups (p = 0.912). A one-way ANOVA was used for analyzing the histological data following confirmation of homogeneity of variance between groups using Levene’s tests and confirmation of normality using Anderson-Darling tests. An α level of 0.05 was used for all comparisons.

## Data Availability

The datasets generated and analyzed during the current study are available from the corresponding author on reasonable request.
